# Gold Enhanced Graphene-Based Photodetector on Optical Fiber with Ultrasensitivity over Near-Infrared Bands

**DOI:** 10.3390/nano12010124

**Published:** 2021-12-30

**Authors:** Wenguo Zhu, Songqing Yang, Huadan Zheng, Yuansong Zhan, Dongquan Li, Guobiao Cen, Jieyuan Tang, Huihui Lu, Jun Zhang, Zhijuan Zhao, Wenjie Mai, Weiguang Xie, Wenxiao Fang, Guoguang Lu, Jianhui Yu, Zhe Chen

**Affiliations:** 1 Guangdong Provincial Key Laboratory of Optical Fiber Sensing and Communications, Department of Optoelectronic Engineering, Jinan University, Guangzhou 510632, China; zhuwg88@163.com (W.Z.); youngsongqing@sina.cn (S.Y.); zhenghuadan@126.com (H.Z.); ccdbys@163.com (J.Z.); 2 Key Laboratory of Optoelectronic Information and Sensing Technologies of Guangdong Higher Education Institutes, Department of Optoelectronic Engineering, Jinan University, Guangzhou 510632, China; 18507735727@163.com (Y.Z.); lidongquan1990@163.com (D.L.); tangjiey@163.com (J.T.); thuihuilu@jnu.edu.cn (H.L.); 3 Siyuan Laboratory, Guangzhou Key Laboratory of Vacuum Coating Technologies and New Energy Materials, Guangdong Provincial Engineering Technology Research Center of Vacuum Coating Technologies and New Energy Materials, Department of Physics, Jinan University, Guangzhou 510632, China; cgb1692190612@stu2020.jnu.edu.cn (G.C.); zz.1987@163.com (Z.Z.); wenjiemai@gmail.com (W.M.); wgxie@email.jnu.edu.cn (W.X.); 4 Key Laboratory of Visible Light Communications of Guangzhou, Jinan University, Guangzhou 510632, China; 5 Science and Technology on Reliability Physics and Application of Electronic Component Laboratory, China Electronic Product Reliability and Environmental Testing Research Institute, Guangzhou 510610, China; fangwx@ceprei.com (W.F.); luguog@126.com (G.L.)

**Keywords:** graphene, photodetector, ultrasensitivity

## Abstract

Graphene has been widely used in photodetectors; however its photoresponsivity is limited due to the intrinsic low absorption of graphene. To enhance the graphene absorption, a waveguide structure with an extended interaction length and plasmonic resonance with light field enhancement are often employed. However, the operation bandwidth is narrowed when this happens. Here, a novel graphene-based all-fiber photodetector (AFPD) was demonstrated with ultrahigh responsivity over a full near-infrared band. The AFPD benefits from the gold-enhanced absorption when an interdigitated Au electrode is fabricated onto a Graphene-PMMA film covered over a side-polished fiber (SFP). Interestingly, the AFPD shows a photoresponsivity of >1 × 10^4^ A/W and an external quantum efficiency of >4.6 × 10^6^% over a broadband region of 980–1620 nm. The proposed device provides a simple, low-cost, efficient, and robust way to detect optical fiber signals with intriguing capabilities in terms of distributed photodetection and on-line power monitoring, which is highly desirable for a fiber-optic communication system.

## 1. Introduction

As an optical to electrical signal converter, the photodetector is an indispensable element in every fiber-optic communication system [1,2,3,4]. Integrating photodetectors into optical fibers to achieve all-fiber photodetection is highly desirable, owing to the intriguing capabilities of distributed photodetection and on-line power monitoring, as well as their structural compactness [3,4]. Conventional photodetectors are based on Si, Ge; semiconductors cannot be perfectly integrated with optical fiber platforms, because of their different geometries and structures. The recently developed two-dimensional (2D) materials provide an opportunity for the all-fiber photodetector (AFPD) [3,4,5,6,7]. Several AFPDs are fabricated based on 2D materials, which suffer from low responsivity and visible operation wavelength [3,4,8]. A high responsivity AFPD-covering telecom band is missing from the literature.

Graphene holds great promise for novel photonic devices including photodetectors [9,10,11,12,13,14,15]. With a high carrier mobility (>200,000 cm^2^ V^−1^ s^−1^), graphene-based photodetectors (GPD) can potentially operate at speeds >500 GHz [1]. The gapless band structure and linear dispersion of electrons enable graphene to be an ultra-broadband photodetector, which is capable of operating over the wavelength range from visible to far infrared [16,17]. However, the intrinsic low optical absorption and short lifetime of the minority carrier in graphene limit the responsivity of the pristine graphene photodetector [1,16]. Absorption materials, such as PbS quantum dots [18], Au nanoparticles [19,20], up-conversion nanoparticles [21,22], perovskite [23], and carbon nanotubes [17], have been demonstrated to significantly enhance the photoelectric responsivity of graphene. However, most of the absorption materials have narrow absorption bandwidths, which narrow the operation wavelength range [18,19,20,21] and will also greatly slow down the response speed of the GPD, because the contact of the absorption material will largely increase electron scattering, thus greatly reducing the conductivity of the graphene. With the advantage of maintaining the outstanding optoelectronic properties of the graphene, a graphene-covered waveguide provides an alternative efficient way to enhance the optical interaction and absorption of graphene by propagating light along the graphene layer [24]. Besides, such waveguide photodetectors have other inherent advantages, such as easy integration and compatibility with the silicon platform. However, the photoelectric feature of silicon limits the operational bandwidth of the waveguide photodetector in the infrared wavelength region [10]. By now, the responsivities of waveguide photodetectors are smaller than 1 A/W at the wavelength of ~1550 nm [10,16,24,25].

To address this problem, we designed an AFPD by assembling a monolayer of graphene onto a side-polished fiber (SPF) with surface plasmon enhancement. SPF has become a versatile all-fiber platform for various electro-optic devices [26,27,28,29]. The evanescent coupling of guiding light in fiber cores to overlaid material uses SPF to find applications ranging from broadband polarizers [27], mode-locked fiber lasers [28], electro-optical modulator [29], to optical sensors [30,31]. In the AFPD, a 448 nm thick PMMA/graphene film layer over the SPF acts as a high-refractive-index waveguide so as to achieve a highly efficient optical field coupling from the fiber core to the PMMA film, thus enhances the interaction between the light and the graphene. A microscale multiple of interdigitated metal fingers is deposited on the top of the graphene film as an electrode for the collection of the photocurrent. Moreover, our numerical simulation finds that the Au film can enhance light absorption over a broadband region. Thus, a giant responsivity as high as 5.7 × 10^4^ A/W and an external quantum efficiency of 4.6 × 10^6^% are achieved at the wavelength of 1520 nm for such graphene AFPDs. Additionally, it has been found that, over a broadband region of 980–1620 nm, the graphene AFPD have high responsivity (exceed 1 × 10^4^ A/W).

## 2. Materials and Methods

Methods of device fabrication. A conventional single-mode fiber (SMF-28e) was polished using a wheel side-polishing technique. A monolayer graphene film (Chengdu Organic Chemicals Co. Ltd., Chinese Academy of Sciences, Chengdu, China) was grown on a 35 µm thick copper foil via low-pressure thermal CVD. In order to transfer the graphene onto the SPF, the poly (methyl methacrylate) (PMMA) was spin-coated onto the graphene film with a thickness of ~448 nm as a supporting layer. The film was then floated on the ammonium persulphate aqueous solution so as to etch the copper. After etching Cu foil, the monolayer graphene sits below the PMMA floating on the surface of the water. In order to remove the impurities, we transferred the graphene/PMMA film through a PET substrate of deionized water and soaked for 30 min. Then, we used a PET substrate to fish up graphene/PMMA film, turned over the PET substrate, and put the graphene/PMMA film back into unpolluted deionized water. At that time, the monolayer of graphene was located above the PMMA. Finally, the graphene/PMMA film was transferred over the SPF. The multiple interdigitated metal fingers with a thickness of 50 nm of Au were deposited on the top of the graphene using physical vapor deposition method with a metal mask. Using the same transfer technique, the hole mobility of CVD-grown graphene transferred by PMMA film is approximately 422.4 cm^2^ V^−1^ s^−1^ according to previous research [32].

For photoresponse characterization, four different laser sources were used to generate lights with wavelengths of 980 (Pump-LSB-980-500-SM, OPEAK, Tianjin, China), 1310 (SOF-131-D, ACCELINK, Wuhan, China), 1480 (Pump-LSB-1480-350-SM, OPEAK, Tianjin, China), and 1520–1620 nm (TSL-550, Santec, Japan). A VOA was used to adjust incident power, while a PC was used to optimize the photocurrent. The PC was fixed during measurements. The photocurrent was measured using a Keithley-2450 sourcemeter (Cleveland, OH, USA). In the temporal photoresponse measurement, a modulated 1550 nm laser source (TSL-550, Santec, Japan) was amplified by EDFA (CEFA-C-PB-HP-PM-37-NL0-OM1-B203-FA-FA, Keopsys, France), with an output power of 1 mW.

## 3. Results and Discussion

**Figure 1:**
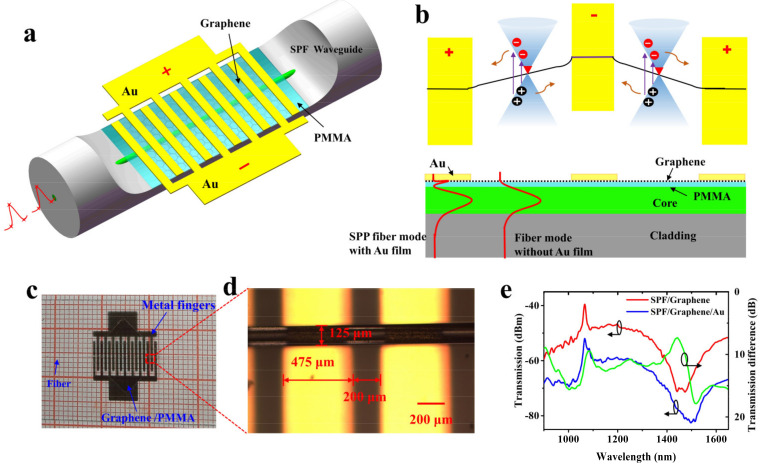
Graphene-based all-fiber photodetector. (**a**) Schematic of the graphene-based AFPD, in which a graphene/PMMA film is transferred onto an SPF and deposited using multiple interdigitated metal fingers. (**b**) (bottom) Lateral view of the AFPD, where the fiber modes in the regions with and without Au films are different. The light field is enhanced near graphene using the SPP fiber mode with Au film. (top) Incident photons create electron–hole pairs in graphene, which are separated by the applied bias field, and form photocurrent. (**c**) A photographic image of the fabricated device on a millimeter square paper. (**d**) Microscope image of the interdigitated metal electrode. (**e**) Transmission spectra of SPF covered with graphene (red line) and SPF with a graphene and Au electrode (blue line), and their difference (green line).

**Figure A1:**
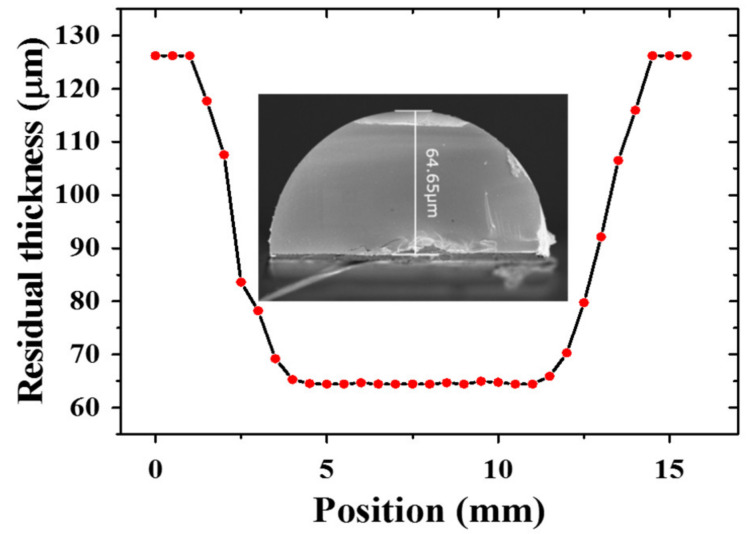
The residual thickness of the SPF. The uniform region of the SPF is about 8 mm thick, with two transition regions 3 mm in length. The inset shows the SEM image of the cross-section, indicating that the residual thickness of the uniform region is 64.65 μm. Thus, part of the fiber core (1.85 μm) is polished.

**Figure A2:**
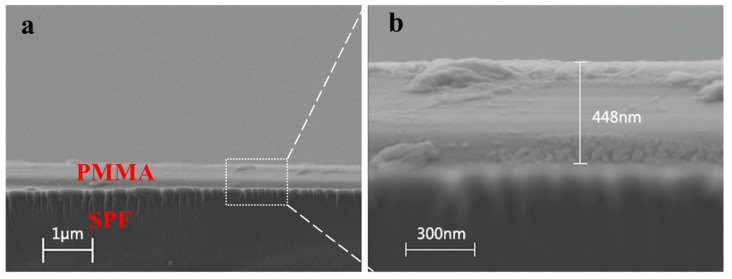
The thickness of PMMA. The SEM images of PMMA in 1 μm (**a**) and 300 nm (**b**) scales, showing the PMMA thickness is approximately 448 nm.

**Figure A3:**
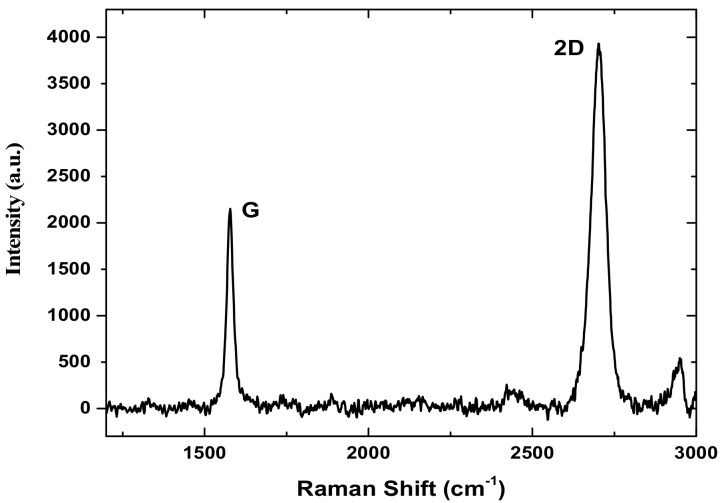
Raman spectrum of graphene deposited on 487 nm PMMA/SPF with 514 nm wavelength laser excitation. G and 2D bands are located at 1578.2 and 2701.9 cm^−1^, respectively. I_2D_/I_G_ = ~1.83.

The structure of the graphene-based all-fiber device is schematically illustrated in Figure 1a. An SPF with a side-polished region was made from a single-mode fiber (SMF-28e) using a wheel side-polishing technique [31]. The length and residual thickness of the side-polished region were ~8 mm and ~64.65 µm, respectively (Appendix A Figure A1). Part of the fiber core was polished. The SPF was placed on a slide glass. A high-quality large-area monolayer of graphene was prepared using chemical vapor deposition (CVD) and spin-coated with a thin layer of PMMA before being transferred onto the polished region of the SPF. The PMMA with a refractive index of *n* = 1.49 and a thickness of ~448 nm (Appendix A Figure A2) drew the evanescent tail of the propagating mode out of the core, thus enhancing the interaction between the light and the graphene. Multiple interdigitated metal fingers were deposited on the top of the graphene layer by physical vapor deposition. A total of 5 nm Cr and 45 nm Au were deposited subsequently. Figure 1b shows the photodetection schematic of graphene-based AFPD. In the side-polished region, the guiding light in the fiber core coupled evanescently with graphene. The field modes in regions with and without Au films were different. The light field beneath the Au film was confined and enhanced, resulting in the enhancement of the light–graphene interaction. Using applied bias voltage, the photo-excited electron–hole pairs were separated, and effective photocurrent was generated, as shown in Figure 1b. Figure 1c shows the photographic image of the fabricated device. The interdigitated electrode was imaged using a microscope. As shown in Figure 1d, the spacing between the metal fingers was 200 μm with a finger width of 475 μm. The metal fingers can not only collect photocurrent but also enhance the absorption of light, thus heightening the responsivity of the AFPD. The interaction length of the side-polished region is estimated to be 8 mm, while a 10 × 10 mm^2^ monolayer of graphene covers the side-polished section. The monolayer property of the graphene was confirmed by the intensity ratio of the 2D and G peak Raman spectra [31] (Appendix A Figure A3). Figure 1e compares the transmission spectra of the SPF covered with graphene and the SPF with the graphene and interdigitated electrode. The difference between the transmission spectra is depicted by the green line, from which one finds that the light absorption increases when the interdigitated electrode is deposited. The Au film induced an absorption increase over a broadband region with a dip around 1518 nm.

**Figure 2:**
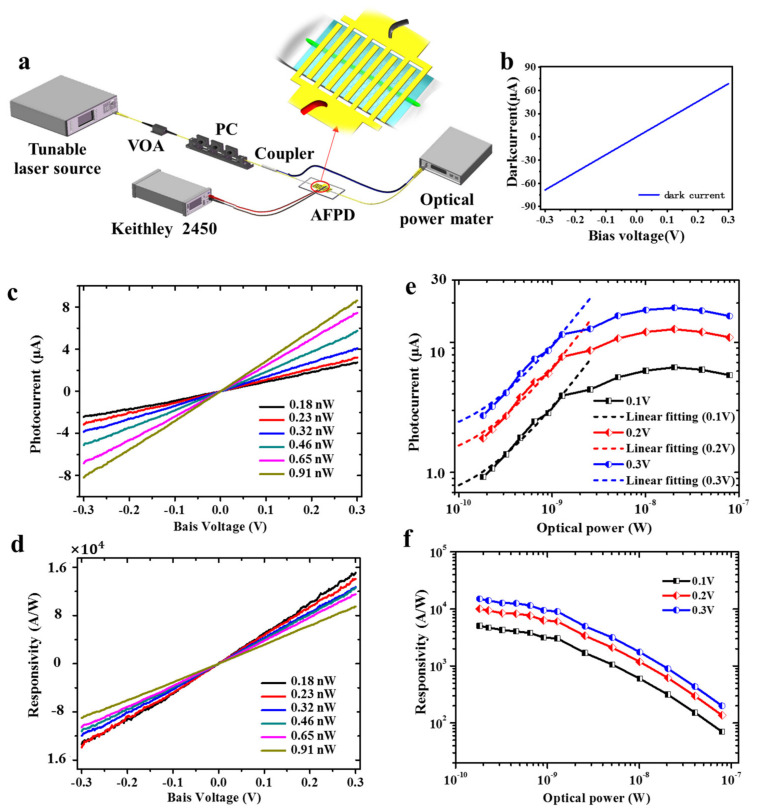
Photoresponse of the AFPD photodetector. (**a)** Experimental setup for the photoresponse characterization. (**b**–**d**) Darkcurrent: (**b**) photocurrent and (**c**) responsivity (**d**) of the AFPD as functions of bias voltage for different light power at the wavelength of 1,530 nm. (**e**,**f**) Photocurrent (**e**) and responsivity (**f**) of the AFPD changing with the light power for *V*_bs_ = 0.1, 0.2, and 0.3 V, respectively. The photocurrents below 1 nW are fitted linearly (dotted lines).

Figure 2a shows the experimental setup for the photoresponse measurement. A 1530 nm light from a tunable laser is guided by single-mode fibers, passing through a variable fiber optic attenuator (VOA) and a polarization controller (PC) successively. The VOA is used to adjust the incident power, while the PC is used to optimize the photocurrent, which is fixed during measurements. Then, a 3 dB coupler separates the incident power into double arms. One arm is used for power calibration, the other is launched into the AFPD device. The generated electrical signal is collected and analyzed using a SourceMeter (Keithley-2450, Cleveland, OH, USA).

Figure 2b shows the changes in photocurrent (*I*_ph_ = *I*_light_ − *I*_dark_) with the bias voltage (*V*_bs_) for different light power. The photocurrents vanish when *V*_bs_ = 0, as predicted. To operate with zero bias voltage, an asymmetric metallization scheme should be used to break the mirror symmetry of the built-in potential profile within the SPF [1]. The bias voltage creates a photocurrent under illumination. The photocurrents are linearly proportional to the bias voltage. As shown in Figure 2d, under a certain fixed bias voltage, the photocurrent increases linearly with the incident power at the beginning, and then becomes nonlinearly (much slower) after 1 nW. Thus, 1 nW can be considered as the saturation power. The photocurrents below 1 nW are fitted linearly with correlation coefficients of 0.986 ± 0.003. The slopes for the cases of 0.1, 0.2, and 0.3 V are 2666.1, 5305.7, and 7879.8 A/W, respectively, with corresponding intercepts of 0.53, 1.08, and 1.66 μA. The saturation property of graphene has been widely used in mode-lock lasers and nonlinear optics [28].

**Figure A4:**
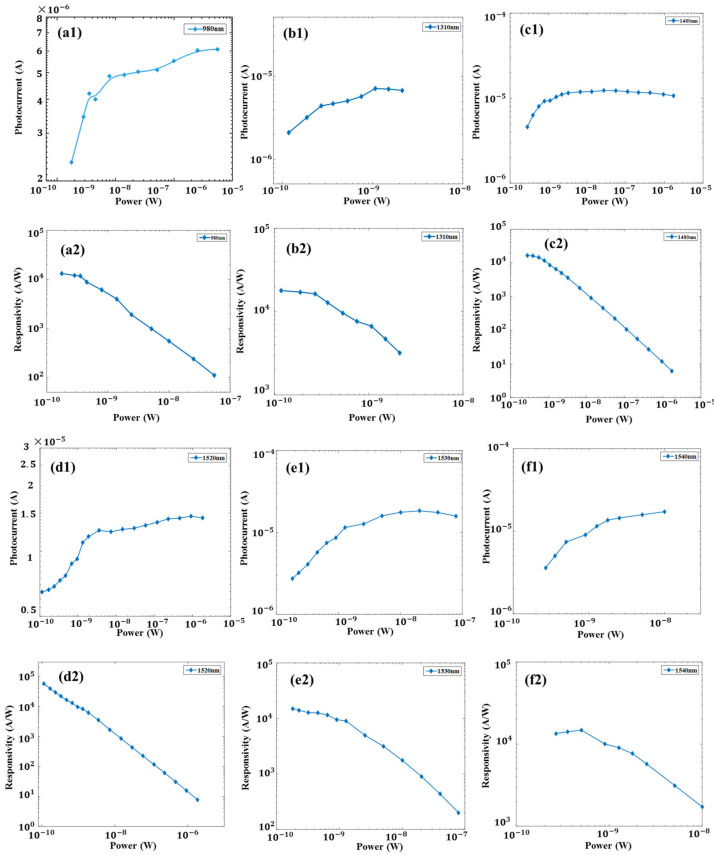
Photoresponse in the wavelength range of 980–1540 nm. The dependences of the photocurrents (**a1**–**f1**) and responsivities (**a2**–**f2**) on the incident optical power, (**a1**,**a2**) are in the wavelength of 980 nm, (**b1**,**b2**) are in the wavelength of 1310 nm, (**c1**,**c2**) are in the wavelength of 1450 nm, (**d1**,**d2**) are in the wavelength of 1520 nm, (**e1**,**e2**) are in the wavelength of 1530 nm, (**f1**,**f2**) are in the wavelength of 1540 nm.

**Figure A5:**
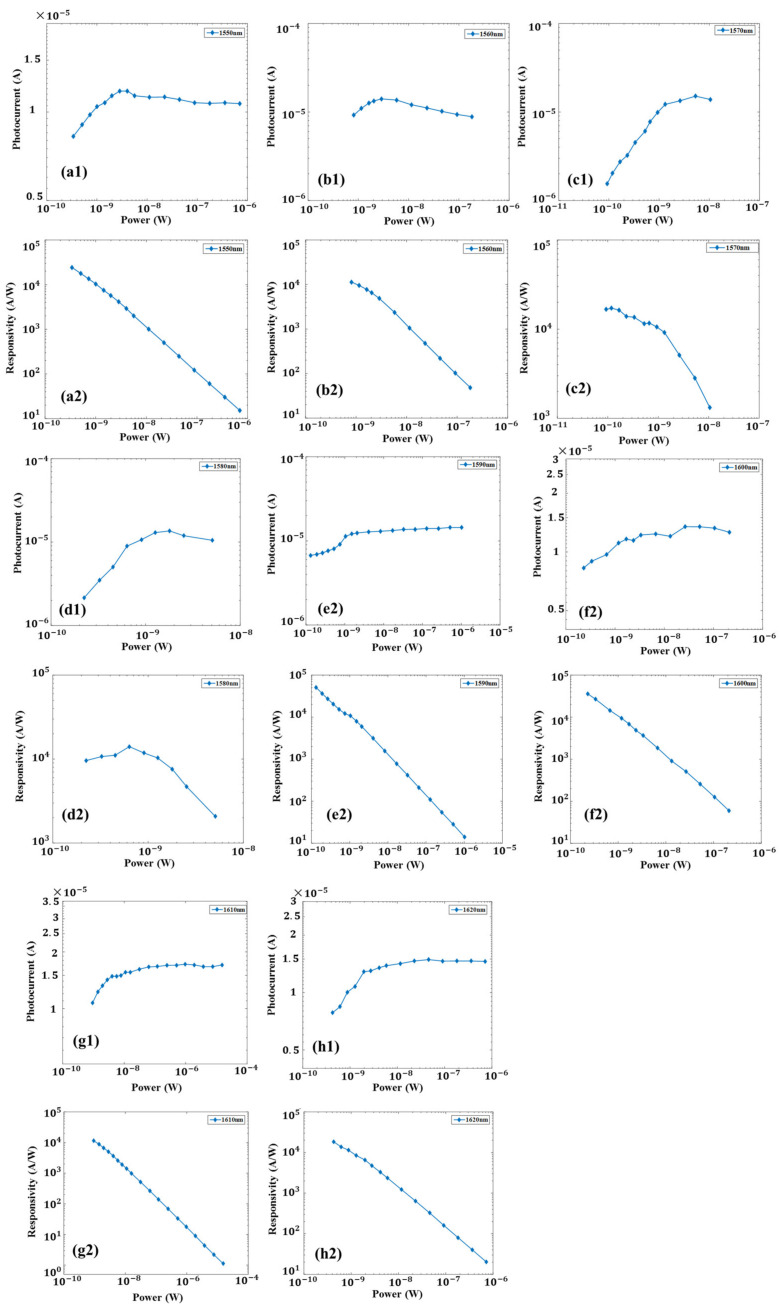
Photoresponse in the wavelength range of 1560–1620 nm. The dependences on incident optical power of the photocurrents (**a1**–**h1**) and responsivities (**a2**–**h2**). The wavelength interval of (**a**–**h**) is 10 nm.

The responsivities (*R* = *I*_ph_/*P*_in_) are calculated and shown in Figure 2c,e. The responsivities possess a linear relationship with the bias voltage. Below the saturation power, the responsivity is nearly independent to the incident power for each bias voltage. The responsivity is up to 1.5 × 10^4^ A/W for *P_in_* = 0.18 nW under the bias voltage of *V*_bs_ = 0.3 V. The external quantum efficiency (EQE) is calculated as 1.2 × 10^6^% according to its definition EQE = *Rhc*/*e*λ, where *h* is the Planck constant, *c* is the light velocity in vacuum, *e* is the electron charge, and *λ* is the light wavelength [2]. The extra-high EQE indicates the photocarrier multiplication process in graphene [33,34]. The maximum responsivity obtained experimentally was 5.7 × 10^4^ A/W (EQE = 4.6 × 10^6^%) for 1520 nm light with a power of 0.11 nW (Appendix A Figure A4). For a 1550 nm incident light, the responsivity is as high as 2.5 × 10^4^ A/W (Appendix A Figure A5). These responsivities are comparable with those of a PbS quantum dot-based photodetector around 1550 nm, which suffers from a slow response time (2 s without reset electric pulse) and air-instability [17]. The 1550 nm responsivity of our AFPD is ~10^9^ times larger than that of a graphene photodetector with an interdigitated metal electrode [1] and ~300 times larger than that of graphene photodetector with plasmon enhancement [35].

**Figure A6:**
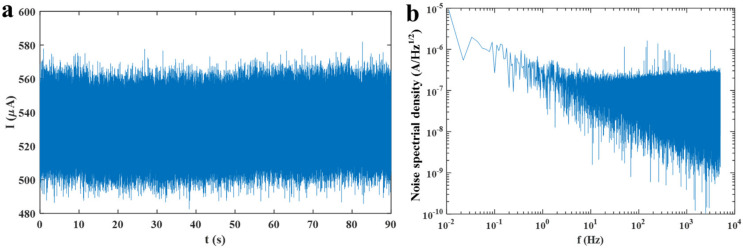
Noise level determination. (**a**) The dark current waveform of the graphene AFPD for *V*_ds_ = 0.3 V corresponding to the same conditions in which we measured its photoresponsivity. (**b**) Noise–power density changing with frequency.

To determine the sensitivity of our photodetector, we measured the noise in the dark current. The frequency-dependent noise spectral density of the photodetector *S_n_* is given under a 0.3 V bias voltage (Appendix A Figure A6). The noise equivalent power (NEP) was found to be ~4 × 10^−12^ W Hz^−1/2^ at 1 Hz, with *S_n_* = ~10^−7^ AHz^−1/2^. The specific dectectivity is defined as D^*^ = *RA*^1/2^/*S_n_*, where *A* is the effective area of the photodetector, estimated as 8 μm × 8 mm = 6.4 × 10^−8^ m^2^. Thus, D^*^ is calculated as 6.3 × 10^9^ Jones.

**Figure 3:**
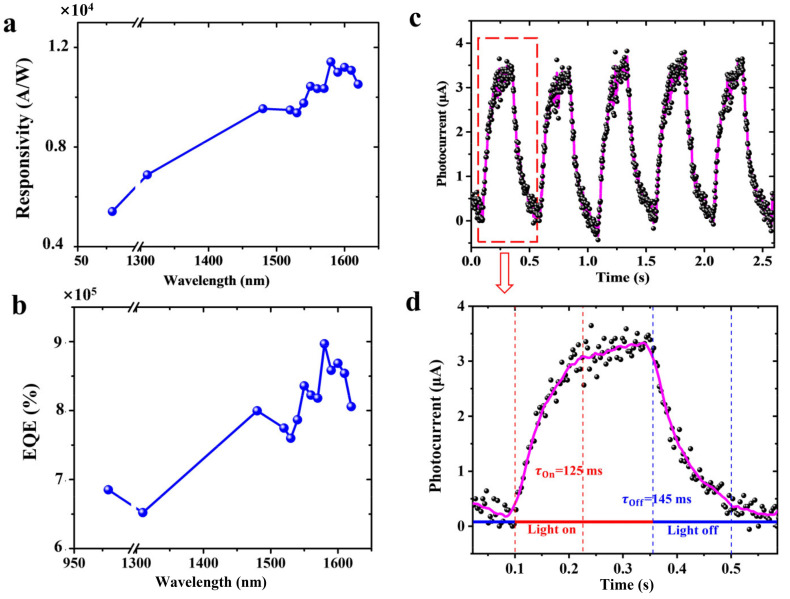
Broadband and transit response of the AFPD. Responsivity (**a**) and EQE (**b**) of different wavelengths when the incident power is fixed at 1 nW. (**c**) Time-dependent photocurrent over a 5-period on–off operation at 1550 nm. (**d**) Zoomed-in view of photocurrent determining the rise and fall times as 125 and 145 ms, respectively.

Response time is another key figure of merit for photodetectors and is also relevant in revealing the physical mechanism of the device operation. Figure 3c shows the photocurrent of the photodetector pumped by a square-modulated 1550 nm laser. The pumped power is 1 mW, which induces a photocurrent of 3.5 μA under a bias voltage of 0.3 V. The photocurrent is repeatable. As shown in Figure 3d, the rise and fall times are estimated as 125 ms and 145 ms, respectively. The transit response can be improved by shortening the channel width of the electrode and increasing the PMMA quality, which will reduce the transit time of carriers and reduce the charged impurity scattering from the PMMA.

Characteristics of the broadband photoresponses of our device over the near-infrared range (980, 1310, 1480, 1520–1620 nm) were investigated using multiple laser sources. The photocurrents and responsivities for various wavelengths are measured for incident power values ranging from ~0.1 nW to ~1 μW, when the bias voltage is fixed at 0.3 V. The measured results can be found in the Appendix A. Due to the broadband properties of graphene, the maximum responsivities exceeded 1 × 10^4^ A/W for all the measured wavelengths. This high response covers the transmission O (1310 nm), S (1480, 1520 nm), C, and L bands (1530–1620 nm) of optical fiber. Figure 3b depicts the responsivity of the AFPD changing with the wavelength at a fixed incident power of 1 nW. The responsivity increases with the wavelength, reaches a peak at 1580 nm, and then decreases gradually. The responsivity is relatively uniform in telecom C band (1530 to 1565 nm), where the responsivities are between 9370 and 10,340 AW^−1^, with a relative change smaller than 10%.

The EQE of the AFPD are calculated for different wavelengths under a 1 nW illumination. In the wavelength region of 980–1620 nm, the EQE is larger than 6 × 10^5^%, as shown in Figure 3b. The maximum EQE is up to 8.9 × 10^5^%, obtained at 1580 nm. The extra-high EQE indicates the photocarrier multiplication process [34].

**Figure 4:**
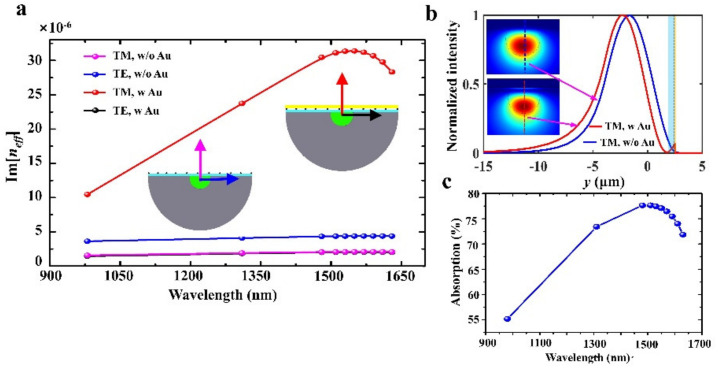
Absorption enhancement by surface plasmon. (**a**) Image parts of the effective refractive indexes of TE and TM guiding modes with and without Au film. (**b**) Normalized intensity of TM mode along the y-axis for the cases with and without Au film. Insets show the mode distributions in the cross plane. (**c**) TM-mode absorption of the AFPD with effective lengths of 5.63 mm region with Au film and 2.37 mm region without Au film.

The ultrasensitive, graphene-based AFPD benefits from the well-designed structure. The thin PMMA layer and integrated metal electrode play key roles in the enhancement of responsivity. To confirm this, mode analyses are performed using Comsol Multiphysics with and without a 50 nm Au film. As shown in Figure 4a, without the Au film, the TE mode is coupled more efficiently with graphene, resulting in a larger image part of the effective refractive index Im[*n_eff_*] than TM mode. However, with the Au film, the TM mode couples more efficiently with coated materials, leading to a much larger Im[*n_eff_*]. This is caused by the light field confinement of the Au film for TM mode [36]. The fundamental TM modes for the cases with and without the Au film are shown by the insets of Figure 4b, where the normalized intensities along the *y*-axis are plotted. The enhancement of the electric field around the Au film is clearly shown in Figure 4b. It is worth noting that the Au enhancement is over a broadband region.

The AFPD contains regions with and without Au films. The effective lengths of regions with and without Au films are 5.63 and 2.37 mm, respectively. The absorption of the AFPD can be estimated by 1-exp{4πIm[neffw/o]Leffw/o+4πIm[neffw/o]Leffw/o}, where the superscript *w* and *w*/*o* denote the regions with and without Au film, respectively. The calculated absorption of the AFPD is given in Figure 4c. The absorption of the TM mode has a peak at 1510 nm, which is in good agreement with the transmission dip at 1518 nm of the spectrum outputted from the AFPD and comparable with the experimentally measured responsivity peak of 1580 nm. 

**Figure A7:**
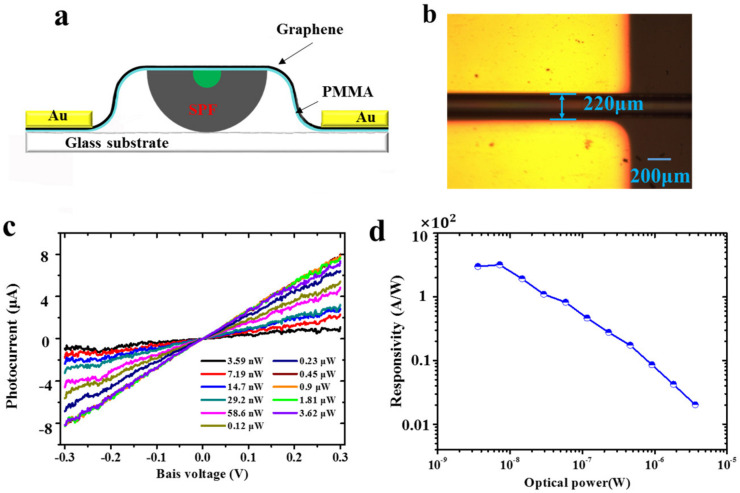
Structure and photoresponse of a photodetector with parallel electrodes. (**a**) A lateral view of the photodetector with parallel electrodes. (**b**) A microscope image of the parallel electrodes. (**c**) Photocurrent changing with the bias voltage for different optical power levels. (**d**) Dependence of responsivity on optical power.

The light field confined around the Au film plays a key role in the responsivity enhancement. A graphene-based photodetector was fabricated with parallel electrodes, as shown in Appendix A Figure A7. The distance between the electrodes is 220 μm, comparable with the spacing (200 μm) between the metal fingers of the interdigitated electrode. However, there is no light-field-enhancement phenomenon in the new electrode structure, as there is no Au film on the SPF. Thus, the light absorption decreases, which will lower the responsivity. The measured results show that the maximum responsivity is 319 A/W at the wavelength of 1530 nm, which is 47 times smaller than that of the AFPD with interdigitated electrode (1.5 × 10^4^ A/W).

**Figure A8:**
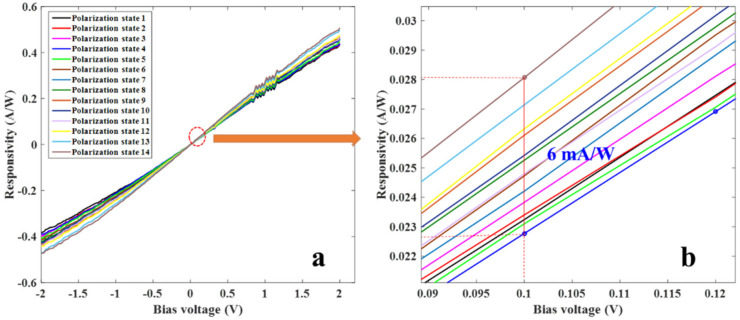
Polarization-sensitive responsivity. (**a**) The dependence of responsivity on bias voltage for different polarization states. (**b**) The zoomed-in view of responsivity for bias voltage values in the range of 0.08 to 0.115 V.

From Figure 4a, one finds that the Im[*n_eff_*] values for the TE and TM modes are different owing to the circular asymmetry of SPF. Thus, the photoresponse of the AFPD is sensitive to the incident polarization, which was verified experimentally (Appendix A Figure A8). By tuning the incident polarization state, the maximum and minimum responsivities are found to be 28 mA/W and 22 mA/W, respectively, with a difference of 6 mA/W (27.3%). The polarization-sensitive property can also be found in photodetectors based on intrinsic anisotropic materials such as black phosphorus [37].

**Figure 5:**
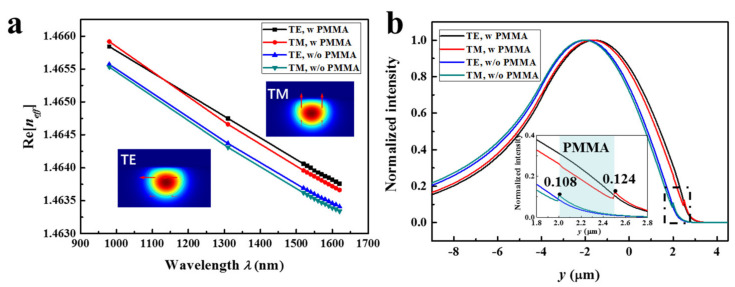
The Full-Vector Finite Element Method (FVFEM) simulation of guiding light between SPF and PMMA. (**a**) The real part of the effective refractive index changing with the wavelength of the TE and TM modes with/without PMMA film. The graphene and Au films are absent in the calculation. Insets show the TE and TM mode distributions in the cross plane for 1550 nm with PMMA. (**b**) Normalized intensities of the TE and TM modes along the y-axis for the cases with and without PMMA. Inset zooms in the region with PMMA film.

Since the PMMA film was physically attached to the SPF, the guiding mode in the SPF/PMMA structure was analyzed to confirm the capability of the light propagating inside the PMMA. Figure 5 compares the TE and TM modes with and without PMMA using FDTD simulation. As shown in Figure 5a, the real part of the effective refractive index Re[*n_eff_*] for both the TE and TM modes with PMMA film are larger than those without PMMA, since the PMMA possesses a larger refractive index than the silica. This indicates that the guiding mode of the fiber core can be effectively coupled into the PMMA film. The normalized intensities along the y-axes in Figure 5b show that the electric fields for both the TE and TM modes are highly efficient couplings of the fiber core and the PMMA film. Without PMMA, the field intensity at the fiber–air interface is 0.108 for TM mode. With the assistance of PMMA, the field intensity increases to 0.124 (15%) at the PMMA–air interface (graphene location). It can be seen that a thin PMMA film can enhance the interaction between the fiber mode and the graphene and, thus, help to promote the photoresponse.

## 4. Conclusions

In conclusion, an Au enhanced graphene-based AFPD with broadband ultrasensitivity over the near-infrared region has been demonstrated. The multiple interdigitated Au fingers enhanced the light–graphene interaction, creating a dramatically enhanced local optical field near the graphene. The extended interaction length of the fiber guiding mode and the coated materials resulted in the high photoresponse. The AFPD can detect light with a power that ranges from low to 0.11 nW, with a responsivity as high as 5.7 × 10^4^ A/W and an external quantum efficiency of 1.2 × 10^6^%. High responsivities (exceeding 1 × 10^4^ AW^−1^) were achieved over a broadband region of 980–1620 nm with a nearly uniform responsivity in telecom C-band. This AFPD is easy to fabrication, low cost, and highly compatible with the current fiber-optical communication system, thus, it facilitates the development of all fiber electrooptical devices.

## Data Availability

The data that support the plots within this paper and other findings of the investigation are available from the corresponding authors upon reasonable request.

## Appendix A

### Appendix A.1. Determination of the Noise Equivalent Power (NEP)

In order to calculate the noise-equivalent power (NEP) of the graphene-based AFPD, we measured the dark current of the device over 90 s, under a bias voltage of *V_ds_* = 0.3 V. The measured results are shown in Figure A6a. The noise–power spectrum was therefore calculated [38] and shown on Figure A6b. At 1 Hz, we obtained *S_n_*(*f* = 1 Hz) = ~10^−7^ AHz^−1/2^. From the responsivity *R* = 2.5 × 10^4^ A/W, we extracted a NEP of ~4 × 10^−12^ W Hz^−1/2^ at 1 Hz.

### Appendix A.2. Photoresponse of the Photodetector with Parallel Electrodes

A photodetector with parallel electrodes was fabricated. As shown in Figure A7a, the SPF on thea glass substrate was deposited by PMMA/graphene. A parallel electrode with a spacing of 220 μm was deposited on graphene using a physical vapor deposition method (see Figure A7b). The photoresponse of the photodetector was measured by a Keithley-2450 SourceMeter (Cleveland, OH, USA). The photocurrent at 1530 nm is shown in Figure A7c for different incident optical power and bias voltage measurements. Figure A7d shows the responsivity changing with optical power for a fixed bias voltage of 0.3 V. The responsivity increases with the decrease of the optical power and remains steady when the power is smaller than 7.19 nW [18,39]. The maximum responsivity is 319 A/W, which is 47 times smaller than that of the AFPD with the interdigitated electrode (1.5 × 10^4^ A/W).

### Appendix A.3. Polarization-Sensitive Photodetector

The polarization-sensitivity property of the AFPD is measured by launching a 1550 nm laser into the device. The polarization state is tuned by the PC. A total of 14 different polarization states were chosen. For each polarization state, the responsivity changing with bias voltage was measured and shown in Figure A8. To find the polarization states with maximum and minimum responsivity, we fixed the bias voltage at 0.1 V and maximized or minimized the photocurrent. From Figure A5b, one finds that, at 0.1 V, the maximum and minimum responsivities are 28 mA/W and 22 mA/W, respectively. A difference of 6 mA/W (27.3%) was found, confirming the polarization-sensitivity property of the AFPD.
